# Case Report: Long-term visual rehabilitation in extreme early-onset axial myopia complicated by retinal detachment

**DOI:** 10.3389/fmed.2026.1951796

**Published:** 2026-09-14

**Authors:** Yue Wu, Wensheng Li

**Affiliations:** 1 Shanghai Aier Eye Hospital, Aier Eye Hospital Group Co., Ltd., Shanghai, China; 2 Shanghai Aier Eye Institute, Aier Eye Hospital Group Co., Ltd., Shanghai, China; 3 Department of Ophthalmology, Tong Ren Hospital, Shanghai Jiao Tong University School of Medicine, Shanghai, China

**Keywords:** case report, pathologic myopia, refractive lens exchange, retinal detachment, visual rehabilitation

## Abstract

Extreme early-onset axial myopia causes profound refractive disability and lifelong vitreoretinal vulnerability. We report approximately 20 years of follow-up in a woman with bilateral myopia of −8.00 diopters (D) at 1 year of age, axial lengths exceeding 30 mm at 6 years and 35 mm before adulthood. At 18 years, severe spectacle-related minification, peripheral distortion, and inconvenience persisted. After discussion of contact lenses, phakic intraocular lenses, and refractive lens exchange, she chose sequential bilateral refractive lens exchange with toric intraocular lenses and a residual myopic target of approximately −4.50 D. Eighteen days after right-eye surgery, she presented with a 3-day history of visual-field obscuration and was found to have a macula-off, multibreak rhegmatogenous retinal detachment. The eye underwent pars plana vitrectomy, endolaser photocoagulation, external cryotherapy, and silicone-oil tamponade; the oil was removed 5 months later. At the final visit, at 21 years of age, the axial lengths were 36.57 mm in the right eye and 36.17 mm in the left eye, and both retinas were attached. Visual acuity through the same-visit subjective refraction was logMAR 0.10 and 0.22 in the right and left eyes, respectively, whereas habitual spectacle-corrected visual acuity was logMAR 0.00 and 0.10. The patient reported improved daily visual function. This case shows that refractive lens exchange can reduce the optical burden of extreme myopic spectacles but does not remove the underlying vitreoretinal risk. It is a cautionary observation rather than a treatment recommendation and underscores individualized etiologic assessment, comparison of alternatives, conservative refractive targeting, peripheral retinal management, and lifelong surveillance.

## Introduction

High myopia before age 7 is uncommon, with prevalence estimates of 0.03%–0.20% in China and Singapore (1). Its etiologic spectrum differs from common school-age myopia; inherited retinal disease, Stickler syndrome, Marfan syndrome, congenital glaucoma, and other syndromic causes should be considered (1). Eyes longer than 35 mm are rarely described as a discrete clinical subgroup and may show posterior globe deformation, myopic maculopathy, and peripheral retinal degeneration that restrict refractive options (2).

Very high-power spectacles can cause marked retinal-image minification, peripheral distortion, and field restriction. Conversely, intraocular refractive surgery in a young, extremely elongated eye entails loss of accommodation, uncertain refractive prediction, and risks of retinal breaks and retinal detachment (3–6). We report an extreme case beginning in infancy, followed by bilateral refractive lens exchange in early adulthood and an early right-eye retinal detachment, with emphasis on long-term functional outcome and risk trade-offs.

## Case description

### Medical history and etiologic assessment

The patient had an unremarkable birth and perinatal history. Family history was negative for high myopia, retinal dystrophy, or other known inherited disorders. Neither parent nor other family members had high myopia, and the parents were not consanguineous. Exact parental refractions were unavailable. Bilateral myopia of approximately −8.00 D was detected at age 1, and spectacles were prescribed. Axial lengths were 26.56 and 26.90 mm at age 3 and 30.02 and 30.06 mm at age 6 in the right and left eyes, respectively. At age 6, she underwent bilateral posterior scleral reinforcement combined with superficial temporal artery diversion at another hospital. The graft material and specific technique could not be verified.

In 2023, pattern electroretinography, multifocal electroretinography, and pattern visual evoked potentials did not show abnormalities diagnostic of an inherited retinal disorder. The patient and her family declined genetic testing. In the absence of full-field electroretinography, comprehensive systemic and genetic assessment, and complete childhood records, the etiology remained undetermined.

At 15 years, a left-eye macular hemorrhage was treated with one intravitreal ranibizumab injection. Axial length exceeded 35 mm in both eyes by 17 years. Ultra-widefield fundus imaging (UWF; Optos plc, Dunfermline, UK) and long-line optical coherence tomography (OCT; Spectralis, Heidelberg Engineering GmbH, Heidelberg, Germany) in July 2022 showed extensive bilateral myopic chorioretinal atrophy, marked posterior-pole curvature abnormalities, and macular retinoschisis. Because contemporaneous fluorescein angiography and optical coherence tomography angiography were not available for the prior left macular hemorrhage, myopic choroidal neovascularization could not be established.

### Preoperative assessment and shared decision-making

Visual acuity was assessed using a 2.5-m logarithmic acuity chart. Source records documented acuity in decimal notation, which was converted to logMAR using logMAR=−log10(decimal acuity). Adult refractions were subjective unless otherwise specified. Childhood records did not document whether cycloplegia was performed, which cycloplegic agent was used, or whether all early refractive values represented spherical equivalents.

On 3 January 2023, optical biometry with the IOLMaster 700 (Carl Zeiss Meditec AG, Jena, Germany) showed axial lengths of 35.63 and 35.46 mm in the right and left eyes, respectively. The corresponding output standard deviations were 27 and 15 μm; the number of repeat scans was not retained. Non-contact tonometry (NCT) values were 13.4 and 12.1 mmHg. Keratometry was 42.72 D at 8° and 44.20 D at 98° in the right eye, and 42.63 D at 178° and 44.31 D at 88° in the left eye. Subjective refraction was −25.00 D sphere with −0.75 D cylinder at 70° in the right eye and −25.00 D sphere with −0.75 D cylinder at 180° in the left eye; corrected visual acuity was logMAR 0.15 and 0.30. Corneal tomography showed apical and thinnest corneal thicknesses of 526 and 521 μm in the right eye and 519 and 515 μm in the left eye, respectively. Anterior chamber depth was 3.34 and 3.27 mm, and endothelial cell density was 3045 and 2,998 cells/mm^2^, respectively. Peripheral retinal degeneration was present bilaterally without an identified break, and prophylactic laser photocoagulation was not performed. Preoperative UWF and vertical long-line OCT are shown in Figure 1.

**Figure 1.**
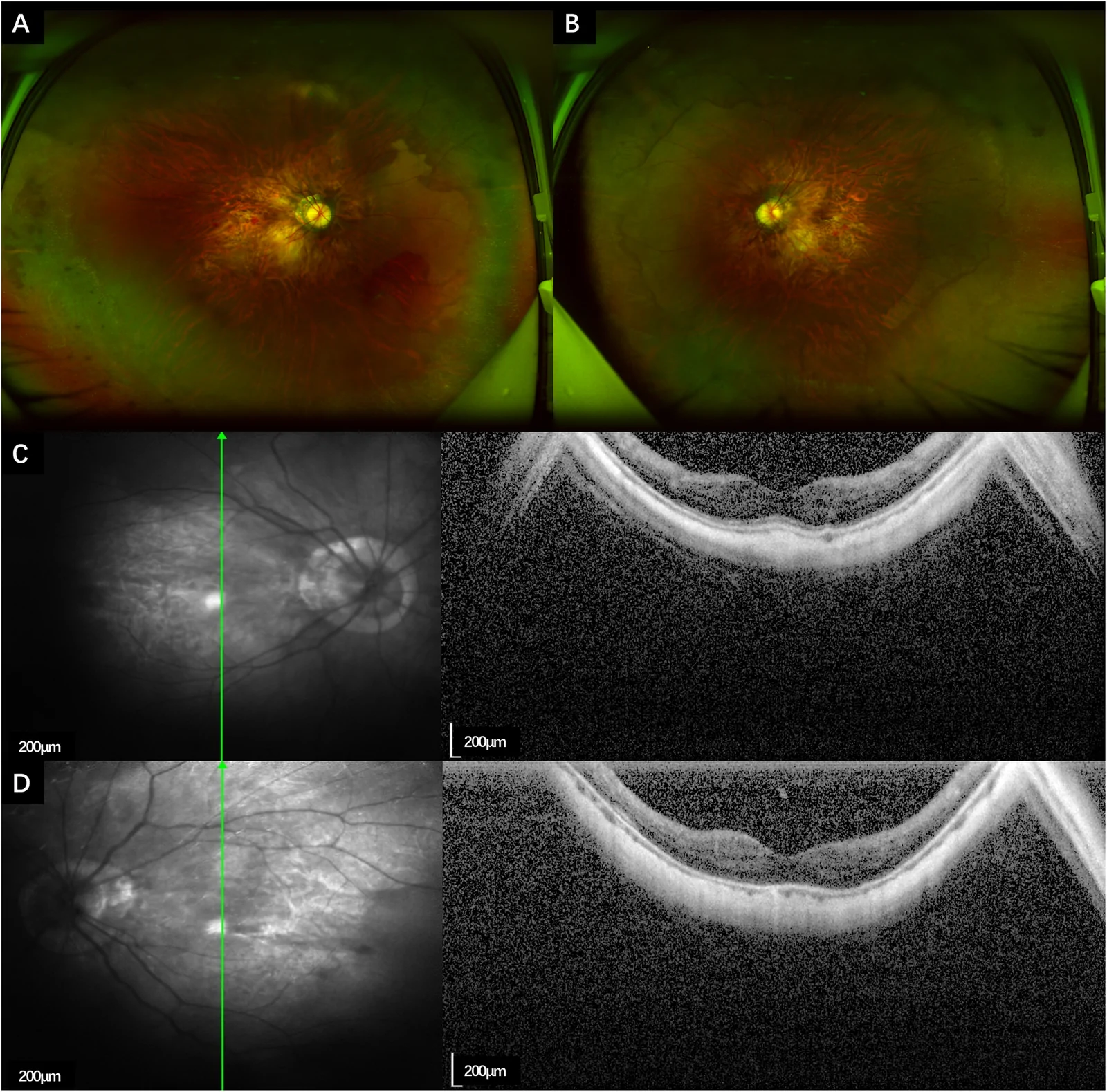
Preoperative bilateral multimodal imaging before refractive lens exchange. **(A,B)** UWF images showing extensive bilateral myopic chorioretinal atrophy. **(C,D)** Vertical long-line OCT scans showing pronounced posterior globe curvature and bilateral macular retinoschisis.

Contact lenses, phakic intraocular lenses, and refractive lens exchange were discussed with the patient and her family. The patient considered long-term contact-lens use insufficiently convenient and understood that a phakic intraocular lens would preserve the crystalline lens and accommodation.

After considering the extreme refractive error, visual quality, lifestyle, and personal preference, she chose refractive lens exchange. She understood the permanent loss of accommodation and the risks of refractive prediction error, intraocular-lens rotation, retinal breaks, and retinal detachment, and accepted continued use of low-myopia spectacles.

## Therapeutic intervention

### Bilateral refractive lens exchange

Intraocular lens (IOL) power and toric alignment were calculated using the ZEISS Z CALC 2 platform (Carl Zeiss Meditec AG, Jena, Germany) with the SRK-T formula and a User Group for Laser Interference Biometry (ULIB)-optimized A-constant of 118.5. Calculation inputs included axial lengths of 35.40 and 35.15 mm, anterior chamber depths of 3.34 and 3.27 mm, and keratometry of 42.73 D at 9° and 44.21 D at 99° in the right eye and 42.49 D at 177° and 44.31 D at 87° in the left eye. No additional axial-length adjustment was applied. A residual myopic target of approximately −4.50 D was selected to reduce the risk of a hyperopic outcome and preserve useful unaided near vision. On 4 January 2023, the left eye underwent clear-lens phacoemulsification with implantation of an AT TORBI 709M toric IOL (Carl Zeiss Meditec AG, Jena, Germany), with powers of −1.00 D sphere and +2.00 D cylinder at a planned axis of 82°; the Z CALC output predicted a postoperative spherical equivalent of −4.60 D. On 11 January 2023, the right eye underwent the same procedure with the same lens model, with powers of −1.50 D sphere and +1.50 D cylinder at 95°; the predicted spherical equivalent was −4.37 D. Neither procedure had a recorded intraoperative complication. The 1-week interval was chosen to reduce postoperative anisometropia and reflected the patient's preference to complete treatment promptly. Postoperative IOL axis was not assessed under dilation, so rotational stability could not be evaluated.

### Right-eye retinal detachment and repair

At the early postoperative review on 12 January 2023, NCT was 14.8 mmHg in the right eye and 19.3 mmHg in the left eye. Visual acuity was assessed through a −4.00 D spherical trial lens in each eye; no manifest refraction was recorded. On 29 January 2023, 18 days after right-eye surgery, the patient presented after 3 days of right-sided visual-field obscuration. NCT was 17.2 and 24.6 mmHg in the right and left eyes, respectively. Neither left-eye NCT reading was documented as repeated, and no IOP-lowering treatment was recorded. Subjective refraction was −4.50 D sphere with corrected visual acuity of logMAR 0.82 in the right eye and −4.25 D sphere with corrected visual acuity of logMAR 0.15 in the left eye. The right retinal detachment extended from approximately 11 to 5 o’clock and involved the macula. Multiple small breaks were present at 10 o’clock, another break at the end of a degeneration area at 5 o’clock, and dry equatorial holes at 2 and 4 o’clock. Breaks and degeneration involved multiple quadrants, with no proliferative vitreoretinopathy (Figure 2). The pre-existing right-eye retinoschitic change could not be assessed separately because of the superimposed neurosensory detachment, whereas mild retinoschitic change remained visible in the attached left macula.

**Figure 2:**
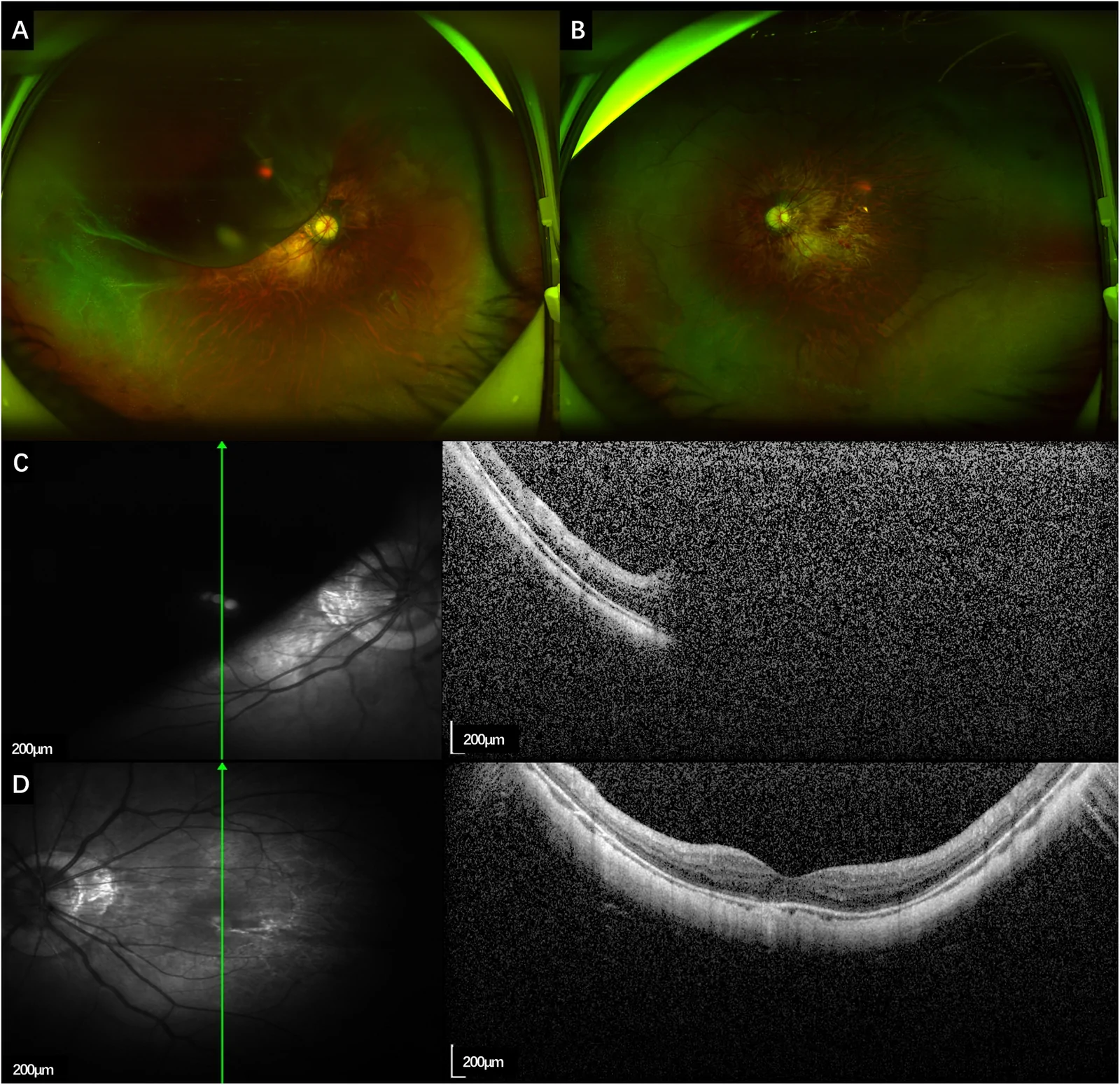
Bilateral multimodal imaging at presentation with right-eye retinal detachment. **(A,B)** UWF images showing macula-off retinal detachment in the right eye and an attached retina in the left eye. **(C,D)** Vertical long-line OCT scans showing right neurosensory retinal detachment, limiting assessment of the pre-existing retinoschisis, and mild persistent retinoschisis in the attached left macula.

The next day, the right eye underwent 23-gauge pars plana vitrectomy, fluid–air exchange, endolaser photocoagulation to the breaks and degeneration, and external cryotherapy to the superotemporal break. No encircling band or scleral buckle was used. Because the detachment involved the macula, with multiple breaks, multiquadrant degeneration, and an axial length exceeding 35 mm, the surgeon selected Oxane 5,700 silicone oil (VRL600; viscosity 5,000–5,900 mPa·s) (Bausch + Lomb, Rochester, NY, USA) for sustained tamponade. Following repair of the right-eye detachment, the left eye received prophylactic laser photocoagulation to superotemporal, inferotemporal, and superonasal degeneration on 31 January 2023. On 5 July 2023, before silicone-oil removal, the right-eye axial length was 36.20 mm by IOLMaster using the silicone-oil-filled-eye measurement mode. Silicone oil was removed later that day. At that visit, right-eye refraction was −1.50 D sphere with −3.25 D cylinder at 180°, and corrected visual acuity was logMAR 0.70.

### Follow-up and outcomes

The left eye underwent neodymium-doped yttrium aluminum garnet posterior capsulotomy for posterior capsule opacification on 15 July 2024. The laser energy and pulse count could not be retrieved from the available records. In February 2025, a right-eye macular hemorrhage occurred. Only structural OCT images were available, and neither fluorescein angiography nor optical coherence tomography angiography was performed. The patient subsequently received three off-label faricimab injections (Vabysmo, F. Hoffmann-La Roche Ltd, Basel, Switzerland). The medical record did not document a comparative rationale for the choice of faricimab. Without angiography, myopic choroidal neovascularization could not be confirmed, and a self-limited lacquer-crack hemorrhage could not be excluded. Follow-up structural OCT documented resolution of the hemorrhage, which was no longer visible by 1 July 2025. At that visit, uncorrected visual acuity was logMAR 0.70, and corrected visual acuity through −4.50 D sphere with −0.75 D cylinder at 130° was logMAR 0.15. At final follow-up, structural OCT showed no active intraretinal or subretinal fluid.

At the final visit on 13 January 2026, approximately 3 years after refractive lens exchange, IOLMaster 700 axial lengths were 36.57 and 36.17 mm, with output standard deviations of 10 and 13 μm in the right and left eyes, respectively. NCT values were 19.2 and 16.1 mmHg. Subjective refraction was −4.50 D sphere with −0.50 D cylinder at 125° in the right eye and −5.00 D sphere with −0.25 D cylinder at 65° in the left eye. Visual acuity through these refractions was logMAR 0.10 and 0.22, whereas habitual spectacle-corrected visual acuity was logMAR 0.00 and 0.10. UWF and long-line OCT showed attached retinas, extensive bilateral myopic chorioretinal atrophy, pronounced posterior globe deformation within the imaged field, and postoperative changes in the right eye (Figure 3). No definite residual macular retinoschisis, intraretinal fluid, or subretinal fluid was evident. At final follow-up, the atrophic and tractional components were graded A3 and T0, respectively, in both eyes according to the ATN classification 7. The neovascular component was not assigned because the previous macular hemorrhagic episodes lacked contemporaneous angiographic confirmation. Because the complete boundary of the posterior globe deformation was not captured, an Ohno–Matsui posterior staphyloma type was not assigned. Follow-up is scheduled every 6 months with retinal evaluation and glaucoma surveillance.

**Figure 3:**
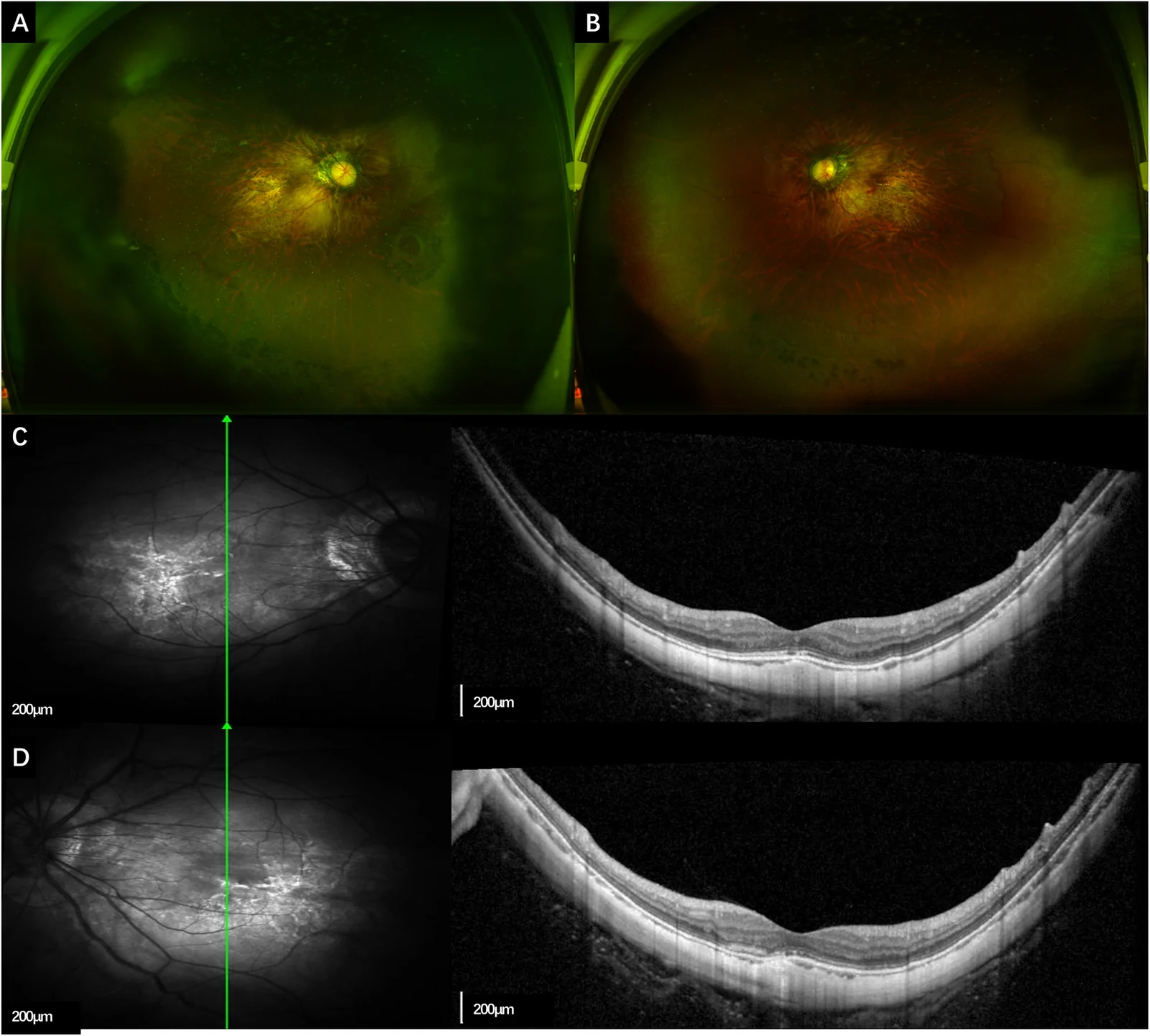
Bilateral multimodal imaging at the final follow-up. **(A,B)** UWF images showing attached retinas, extensive myopic chorioretinal atrophy, and postoperative changes in the right eye. **(C,D)** Vertical long-line OCT scans showing attached maculae, pronounced posterior globe deformation, no definite residual macular retinoschisis, and no intraretinal or subretinal fluid.

### Longitudinal clinical data

Longitudinal ocular data and major clinical events are summarized in Table 1.

**Table 1:** Longitudinal ocular data and clinical events.

Age or date	Refraction (D) OD; OS	Keratometry (D) OD; OS	Axial length (mm) OD; OS	NCT (mmHg) OD; OS	Corrected visual acuity (logMAR) OD; OS	Main event
1 year	−8.00; −8.00	—	—	—	—	Myopia detected; spectacles prescribed.
3 years	−16.50 DS; −17.00 DS	—	26.56; 26.90	—	—	Rapid myopic progression.
6 years	−23.00 DS, −2.00 DC ×40°; −22.00 DS, +1.50 DC ×180°	—	30.02; 30.06	—	0.60^a^; 0.52^a^	Bilateral posterior scleral surgery.
15 years	−27.00 DS; −27.00 DS	—	34.90; 34.69	—	0.30^a^; 0.30^a^	Left macular hemorrhage; one ranibizumab injection.
July 2022	—	42.73 × 9°, 44.21 × 99°; 42.49 × 177°, 44.31 × 87°	35.40; 35.15	—	0.15^a^; 0.30^a^	Bilateral UWF imaging and long-line OCT.
3 Jan 2023	−25.00 DS, −0.75 DC ×70°; x`−25.00 DS, −0.75 DC ×180°	42.72 × 8°, 44.20 × 98°; 42.63 × 178°, 44.31 × 88°	35.63; 35.46	13.4; 12.1	0.15^b^; 0.30^b^	Preoperative assessment.
12 Jan 2023	—^c^; —^c^	—	—	14.8; 19.3	0.70^c^; 0.40^c^	Early postoperative review.
29 Jan 2023	−4.50 DS; −4.25 DS	—	—; —	17.2; 24.6	0.82^b^; 0.15^b^	Right macula-off retinal detachment.
5 Jul 2023	−1.50 DS, −3.25 DC ×180°; −4.50 DS	—	36.20^e^; 35.48	13.4; 12.7	0.70^b^; 0.30^b^	Right silicone-oil removal.
15 Jul 2024	−4.25 DS; −5.00 DS	42.85 × 2°, 44.02 × 92°; 42.48 × 177°, 44.31 × 87°	36.06; 35.79	16.3; 13.5	0.00^b^; 0.52^b^	Left Nd:YAG posterior capsulotomy for posterior capsule opacification.
5 Feb 2025	−4.50 DS, −0.25 DC ×130°; −4.75 DS, −0.50 DC ×30°	42.51 × 180°, 43.79 × 90°; 42.56 × 4°, 44.21 × 94°	36.25; 35.94	—	0.10^b^; 0.22^b^	Right macular hemorrhage; three subsequent faricimab injections.
1 Jul 2025	−4.50 DS, −0.75 DC ×130°; −5.25 DS	42.87 × 174°, 43.72 × 84°; 42.48 × 171°, 44.29 × 81°	36.36; 36.06	16; 15	0.15^b^; 0.22^b^	Right macular hemorrhage resolved; both retinas attached.
13 Jan 2026	−4.50 DS, −0.50 DC ×125°; −5.00 DS, −0.25 DC ×65°	42.88 × 178°, 43.72 × 88°; 42.48 × 175°, 44.20 × 85°	36.57; 36.17	19.2; 16.1	0.10^b^ (0.00^d^); 0.22^b^ (0.10^d^)	Both retinas attached; follow-up every 6 months.

Data are presented as right eye (OD) and left eye (OS). All visual acuity values are reported in logMAR units. An em dash indicates unavailable data. DC, diopter cylinder; DS, diopter sphere.

a Historical corrected acuity; exact correction conditions were incompletely documented.

b Acuity measured through same-visit subjective refraction.

c Acuity was measured through a −4.00 D spherical trial lens in each eye; manifest refraction was unavailable.

d Habitual spectacle-corrected acuity. Discordant Topcon autorefractor keratometry on 12 January 2023 was excluded because its reliability and longitudinal comparability could not be established.

e Right-eye axial length on 5 July 2023 was measured with the IOLMaster before silicone-oil removal using the silicone-oil-filled-eye mode.

## Discussion

This case illustrates that refractive functional improvement and anatomic safety are not equivalent in extreme early-onset axial myopia. The absence of high myopia in the family and the absence of consanguinity do not exclude a genetic or syndromic cause. Because genetic testing was declined and childhood systemic assessment and full-field electroretinography were incomplete, the etiology remains unresolved.

Bilateral extreme myopia during childhood may have caused refractive amblyopia, but historical acuity was also influenced by retinal-image minification from high-power spectacles, peripheral distortion, and posterior-segment disease. After lens exchange, pseudophakic correction reduced vertex distance and spectacle minification. Preoperative spectacle-corrected and postoperative pseudophakic visual acuities are therefore not optically equivalent, and the numerical improvement does not establish that childhood amblyopia was absent or resolved.

For an 18-year-old patient, contact lenses and phakic IOLs should ordinarily be discussed before clear-lens extraction because both preserve the crystalline lens and accommodation. Clear-lens extraction permanently removes accommodation and may add vitreoretinal risk. The choice in this case followed detailed discussion of alternatives and highly individualized shared decision-making; it should not be generalized as routine treatment. The approximately −4.50 D target balanced refractive uncertainty, avoidance of a hyperopic outcome, and unaided near function. The −4.00 D values recorded on 12 January 2023 were trial lenses rather than manifest refractions and therefore cannot serve as an early postoperative formula outcome. The first available postoperative subjective refraction on 29 January coincided with the retinal-detachment presentation, so a formal early prediction error was not calculated. Later refractions are presented as longitudinal refractive change rather than measures of IOL formula error. Because postoperative refraction was not standardized at every time point and toric IOL axes were not assessed under dilation, refractive changes cannot be used to infer formula performance or lens rotation.

Notably, comparison of the 3 January 2023 and 13 January 2026 measurements showed continued axial elongation after pseudophakia. Axial length increased from 35.63 to 36.57 mm in the right eye, an increase of 0.94 mm, and from 35.46 to 36.17 mm in the left eye, an increase of 0.71 mm. This longitudinal change was accompanied by a later myopic shift in the available pseudophakic refractions. Although exact quantification should be interpreted cautiously because measurement-axis variation may occur in extremely elongated eyes with pronounced posterior globe deformation, continued axial elongation into early adulthood may be relevant to refractive target selection and long-term biometric and retinal surveillance.

A 1-week interval reduced the period of marked anisometropia but also limited the opportunity to observe the first eye before operating on the fellow eye. The subsequent complex retinal detachment in the right eye makes this trade-off clinically salient. No preoperative prophylactic laser was applied because degeneration was present without a break; the left eye received prophylactic laser after the severe fellow-eye event. This sequence shows that peripheral retinal management may change with evolving individual risk, but the net benefit of prophylactic laser cannot be determined from one case.

Longitudinal OCT documented bilateral macular retinoschitic change preoperatively. At the retinal-detachment visit, assessment of the pre-existing right-eye retinoschitic change was limited by the superimposed neurosensory detachment, whereas mild retinoschitic change persisted in the attached left macula. At final follow-up, no definite residual macular retinoschisis was evident on the available long-line B-scans in either eye. Although the retinoschitic appearance was no longer evident in the right eye after vitrectomy, this observational sequence cannot establish a causal effect of vitrectomy on the tractional changes.

A systematic review and meta-analysis reported an association between Nd:YAG posterior capsulotomy and increased pseudophakic retinal-detachment risk (8). In this case, capsulotomy was performed only in the left eye approximately 18 months after repair of the right-eye retinal detachment and therefore cannot account for the preceding right-eye event. Nevertheless, it provides an additional reason for continued retinal surveillance in this extremely myopic patient.

Contemporary population and registry data associate younger age and long axial length or high myopia with retinal tear or detachment after lens surgery (3–5). A 10-year cohort found no significant difference in retinal-detachment prevalence between highly myopic eyes with posterior-chamber phakic implantable collamer lenses and nonoperated myopic controls (6). Reports of eyes with extremely long axial lengths have generally involved phacovitrectomy or clear-lens extraction combined with vitrectomy rather than isolated refractive lens exchange in a young adult. Fan et al. reported outcomes after combined phacovitrectomy in such eyes (9), and Zheng et al. reported 4-year outcomes after clear-lens phacoemulsification combined with vitrectomy, including axial lengths approaching 39 mm (10). These populations and vitreous interventions differ from the present case. Direct evidence for isolated refractive lens exchange at age 18 with axial length exceeding 35 mm remains very limited.

Neither macular hemorrhage was confirmed by angiography. In pathologic myopia, hemorrhage from a lacquer crack may resolve without neovascular activity; consequently, the necessity of the prior anti-vascular endothelial growth factor treatments cannot be established retrospectively. If hemorrhage recurs, vascular imaging should, whenever feasible, precede treatment.

Limitations include the retrospective single-case design, incomplete childhood data, and incomplete etiologic testing. Childhood records did not document cycloplegic status, the cycloplegic agent, or whether all early refractive values represented spherical equivalents; correction conditions for some historical acuity measurements were also incomplete. Standardized serial axial-length measurements around the childhood posterior scleral intervention were insufficient for causal assessment. In adulthood, measurement-axis variation in extremely elongated eyes may affect longitudinal comparisons; the relationship between the measurement axis and the deepest posterior deformation could not be verified, and repeat-scan counts were not retained. The right-eye axial length of 36.20 mm on 5 July 2023 was measured before silicone-oil removal in silicone-oil-filled-eye mode; the subsequent decrease to 36.06 mm in July 2024 should not be interpreted as true axial shortening. Serial IOP measurements were obtained by NCT, and no confirmatory applanation measurement was documented for the isolated left-eye readings of 19.3 and 24.6 mmHg. Postoperative toric IOL axes were not assessed under dilation, and contemporaneous vascular imaging was unavailable for the macular hemorrhages.

This report offers a cautionary clinical observation. A patient with extreme early-onset axial myopia may experience meaningful functional improvement after refractive lens exchange, while the underlying retina remains vulnerable to severe events. The case does not support a general treatment recommendation; its practical message is the need to compare alternatives carefully, communicate uncertainty and risk explicitly, use a conservative refractive target, manage peripheral retinal findings individually, and maintain lifelong surveillance.

## Patient perspective

The patient reported that the very high-power spectacles had substantially interfered with study and daily activities. She reported that overall visual function and convenience improved after lens surgery. She understood the experience of right-eye retinal detachment and the persistent long-term risk, accepted continued use of low-myopia spectacles, and agreed to 6-monthly surveillance.

## Data Availability

The original contributions presented in the study are included in the article/Supplementary Material, further inquiries can be directed to the corresponding authors.
